# Editorial: Men, mental health, and suicide

**DOI:** 10.3389/fsoc.2022.1123319

**Published:** 2023-01-16

**Authors:** Anne Cleary, Derek M. Griffith, John Lindsey Oliffe, Simon Rice

**Affiliations:** ^1^UCD Geary Institute for Public Policy and Department of Sociology, University College Dublin, Dublin, Ireland; ^2^Department of Health Management and Policy, School of Health and Centre for Men's Health Equity, Georgetown University, Washington, DC, United States; ^3^School of Nursing, University of British Columbia, Vancouver, BC, Canada; ^4^Department of Nursing, The University of Melbourne, Melbourne, VIC, Australia; ^5^Faculty of Medicine, Dentistry and Health Science based at Orygen, Melbourne, VIC, Australia; ^6^Centre for Youth Mental Health, The University of Melbourne, Melbourne, VIC, Australia

**Keywords:** masculinities, mental health, suicide, suicidal behavior, help-seeking behavior, practices of restrictive emotionality, suicide prevention

Suicide has been identified by the World Health Organisation (2014) as a global health problem disproportionately impacting males. Various psychosocial and neurobiological explanations have been advanced to account for the high rates of suicide in men including unwillingness to engage in mental health help-seeking, lack of gender-sensitive mental health services, impulsivity, alcohol and drug use, and access to and use of lethal means. A cultural/gender perspective has proved insightful in describing how gendered pressures and cultural beliefs about the idealized characteristics and practices of men can heighten the risks for suicide. Cultural/gender explanations are supported by the fact that rates of male suicide vary considerably across the world and fluctuate within societies between sub-groups of men based on socioeconomic status, race/ethnicity, age, sexuality, urban/rural location and or the intersection of these factors. The focus of this Research Topic is on men's mental health, how men respond to mental health challenges and make decisions relating to suicidality. The collection of papers includes contributions from a variety of disciplines and are based on diverse research populations in Europe, the United States and Australia.

Streb et al. begin the discussion by addressing the male excess in suicide rates and the discrepancy between men's low rate of diagnosed depression and high rates of suicide. A possible explanation for this finding is that current depression inventories do not capture typical male symptoms of depression (Martin et al., 2013) and the authors examined gender-specific differences in depression symptoms between men and women in a forensic psychiatric sample. Although externalizing behaviors were similar in both groups, they found a significant relationship between external and internal depression symptoms only in men. This confirms previous research indicating unique symptomology in depressed men characterized by aggressiveness, alcohol use, and risky behavior (Möller-Leimkühler and Mühleck, 2020). These are significant risk factors for suicidal behavior (Rice et al., 2019; Armstrong et al., 2020) and the authors advocate the use of gender-sensitive screening instruments for the early detection of depression symptoms in men.

In terms of identifying suicidal features, practices of restrictive emotionality are identifiable in some, but not all, men. As Gough et al. demonstrate, men disclose difficulties, including anxiety, in safe environments such as therapeutic settings and within intimate partner relationships as well as to male friends. Men's emotion disclosures are also impacted by the ability of others to recognize and empathically engage in such talk in ways that are consistent with the way they create ideals for themselves by using elements of hegemonic masculinity that they value and that they can attain (Griffith, 2022). The analysis highlights how men's anxiety-talk and help-seeking is embedded in and often constrained by interpersonal relationships and social interactions (Evans et al., 2011). Vickery's paper similarly demonstrates the ability of men to disclose emotional problems and the findings imply diversity in how men engage with their mental health and seek help. According to Vickery, the study participants demonstrated that men can embrace alternative patterns of masculinity which enable them to positively engage with their mental health.

Trail et al. continue this exploration of masculinities and health related practices in a rural Australian community. Rural Australian men have a higher risk of dying by suicide and this is frequently linked to their adherence to messages and pressures associated with a tough kind of masculinity and remaining stoic in the face of hardship. Based on qualitative interviews with community workers they identify key areas for understanding suicide risk for these rural men. The contribution of local expressions of masculinity to men's wellbeing and help-seeking behaviors were viewed as key challenges as these men felt pressure to adhere to idealized roles and values. Referenced also were men's disconnections which led to loneliness and isolation that fed their distress and increased suicide risk.

Grigienė et al.'s study focuses on gender identity and suicide risk in Lithuanian men. The findings of their quantitative inquiry showed that higher gender self-definition and higher gender self-acceptance were associated with lower suicide risk and the authors concluded that a stronger gender self-confidence is an important protective factor in relation to male suicide. Accordingly, the authors conclude that while some aspects of masculinity increase suicide risk, therapeutic strategies targeted at bolstering strength-based masculinity are important.

Valdez et al. continue the exploration of different masculinities and the need for place-based health assessments in detailing the challenges of low/no-income Latino men. Based on findings from in-depth interviews with Puerto Rican men living in the United States, their results reveal that participants linked stress to factors such as experiences of racism and prejudice, family relationships, and lack of economic and other resources and also to histories of colonial violence and displacement. The authors propose expanding standardized assessment models to delineate the impact of distinctive historical trajectories to assist in interpreting racial/ethnic health inequities and thus improve interventions.

Two papers explore important sites, the family and school, for the development of gender identity and masculinity values. Cleary explores the implications for men's wellbeing of exposure to family and neighborhood cultures of feeling restriction and the contribution of fathers and father-son relations to these environments. The inquiry, based on a sample of men who made a suicide attempt in adulthood, revealed how exposure to a hegemonic form of masculinity from an early age - such as adhering to masculine values emphasizing strength and emotional stoicism - restricted the respondents in learning about and negotiating the emotional issues of their lives and led to problematic father-son relationships. The study demonstrated challenges for males raised in settings that value rigid forms of hegemonic masculinity and the importance of nurturing father-son relationships for male wellbeing. Wilson et al. also cite the influence of the father on young males in their exploration of teachers' and parents' perspectives on masculinities in a single-sex independent Australian school. Results indicate the impact of Australian masculinity norms and the role of private boys' school cultures in the development of adolescent masculinities but also perceptions of public and private masculinities and the need to upskill parents, particularly fathers, in their capacity to model healthy masculinity norms.

Overall, the Research Topic highlights the existence of diverse masculinities and male mental health-related practices and the need for place-based approaches to prevent suicide. As Balcombe and De Leo suggest, more needs to be known about the complex relationship between mental ill-health, its comorbidities and the biopsychosocial factors that influence suicidality but identifying the contexts in which men can safely share their experiences is key and localized interventions offer the best possibility of success in preventing male deaths by suicide.

## Author contributions

All authors listed have made a substantial, direct, and intellectual contribution to the work and approved it for publication.
